# Ophthalmic Myology

**Published:** 1902-11

**Authors:** 


Ophthalmic Myology. A Systematic Treatise on the Ocular Muscles. By G. C. Savage, M. D., Professor of Ophthalmology in the Medical Department of Vanderbilt University; Author of "New Truths in Ophthalmology." Duodecimo, pp. 595; 61 illustrative cuts and 6 plates. Nashville, Tenn.: Published by the author. 1902.

Dr. Savage has contributed a valuable addition to ophthalmology, giving a résumé of the best known muscular tests and his experience with their limitations. Old theories are put to a rigid test and shown wherein they are faulty by a series of original investigations.

The first chapter shows earnest work and while there may be some discussion about the axis of rotation the rest of the chapter presents the principles of ocular motions with a conciseness and clearness rarely found in any book of this character.

The articles on orthophoria—the usual type and the asthenic—heterophoria, esophoria, exophoria, hyperophoria, cataphoria and cyclophoria, together with less frequent deviations from the normal have never been presented in any better manner. The tests and their histories are well illustrated and their limitations well described. The only criticism would be the omission of certain points on treatment which were perhaps ignored from lack of space.

Theoretically, presbyopia might be deferred by carrying out the ryhthmic exercise proposed in the last chapter, by means of a weak minus lens; but anything as complicated and tedious as the treatment outlined hardly seems practical. R.H.S.

